# Rheumatoid arthritis in an adult patient with mosaic distal 18q-, 18p- and ring chromosome 18

**DOI:** 10.12688/f1000research.11539.2

**Published:** 2018-02-28

**Authors:** Alanna Chau, KH Ramesh, Anand D Jagannath, Shitij Arora

**Affiliations:** 1 Albert Einstein College of Medicine, Bronx, New York City, NY, USA; 2 Department of Pathology, Montefiore Medical Center, Bronx, New York City, NY, USA; 3 Department of Medicine, Montefiore Medical Center, Bronx, New York City, NY, USA

**Keywords:** ring chromosome 18, rheumatoid arthritis, interstitial lung disease

## Abstract

Ring chromosome 18 has a highly variable phenotype, depending on the extent of distal arm deletions. It is most commonly presented as a combination of 18p- and distal 18q- syndrome. IgA deficiency and autoimmune diseases have been previously described in these patients. Seven cases of juvenile rheumatoid arthritis (JRA) have been reported. Here we report the first case of late onset rheumatoid arthritis (RA) in a 32 year old Dominican woman with hypothyroidism, vitiligo, IgA deficiency, interstitial lung disease (ILD), cystic bronchiectasis, and features consistent with ringed 18, 18p- and distal 18q syndrome.  The multiple autoimmune findings in our patient lends further support to the idea of loci on chromosome 18 playing a role in autoimmune disease expression. Late onset RA and ILD in a patient with chromosome 18 abnormalities are novel findings and are additional conditions to be aware of in this population.

## Introduction

Changes in the structure of chromosome 18 are implicated in a number of conditions affecting health and development. 18p- and distal 18q- syndrome has been estimated to occur in 1/50,000 and 1/40,000 live births, respectively. The characteristics of 18p- syndrome are wide ranging and include speech delay, holoprosencephaly spectrum, micrognathia, ptosis, flat nasal bridge, wide mouth with short upper lip, excessive dental caries, large protruding ears, and skeletal abnormalities. 18q- syndrome also presents with a wide variety of clinical features that commonly includes foot anomalies, carp like mouth, midface hypoplasia, cleft palate, cleft lip, inner epicanthal folds, slanted palpebral fissures, narrow or atretic external auditory canals and low set ears. Features common to both syndromes include intellectual disability, short stature, microcephaly, tone abnormalities, seizures, hearing loss and cardiac defects. The phenotypic severity of either condition appears to be correlated with the amount of genetic material affected ^1^.

Ring chromosome 18, or r(18), is a rarer condition that most commonly forms when there is breakage in both chromosome arms, fusion of those breakpoints and the subsequent loss of the distal fragments ^2^. Ring chromosomes can also result from terminal deletions as well as contiguous duplication, with some of these cases demonstrating inversion of these duplications and thus an inv dup del rearrangement mechanism. Individuals with a ring chromosome may have varying levels of mosaicism which is termed as “dynamic tissue mosaicism ^3^. As a result of the inconsistent amount of duplication and hemizygosity of the distal ends, the r (18) phenotype is extremely variable. Clinical characteristics are typically a combination of 18p- and distal 18q- syndrome ^4^. The occurrence of late onset lung disease in probands’ with ring chromosome 18 has not been reported. IgA deficiency and immunological diseases such as type 1 diabetes mellitus (T1DM), juvenile rheumatoid arthritis (JRA), Grave’s disease, hypothyroidism, and vitiligo have been reported in individuals with r(18) ^4– 9^. Here we report the first case of late onset rheumatoid arthritis (RA) associated with mosaic 18p-, distal 18q-, and r(18) in a 32 year old Dominican woman with intellectual disability, hypothyroidism, vitiligo, IgA deficiency, interstitial lung disease (ILD), cystic bronchiectasis, and features consistent with both 18p- and distal 18q- syndrome.

## Case report

### Patient information

The patient was a 32-year-old Dominican woman who presented to the emergency room with fever, hypoxia, wheezing, shortness of breath, cough productive of yellow sputum, and coarse breath sounds. She was hospitalized three months prior for community acquired pneumonia. She was subsequently admitted to the general medicine service for management of presumed healthcare associated pneumonia.

She immigrated with her family to the US from the Dominican Republic 2 years ago. Unfortunately, we have no access to previous medical records and past medical history was obtained from her mother. The patient is the second child of a healthy non-consanguineous couple. At the time of birth, mother and father were 25 and 30 years old, respectively. Prenatal ultrasound was not done. She was born full term with birth weight of 2948 g. At birth, the patient was found to have an abnormal head shape, enlarged heart and heart murmur that resolved by 4 years after taking an unspecified oral medication. Cleft palate and left clubfoot were surgically repaired at age 3 and 4 years, respectively. Dentition was initially normal, however teeth fell early or had extensive caries. At age 9 years skin and hair depigmentation began and she was diagnosed with vitiligo. She is hypothyroid and maintained on levothyroxine. At age 18 years monthly menses began. Over the years hearing has deteriorated, necessitating louder cues to respond. At age 19 years she began to develop morning pain and swelling in her knees. Symptoms progressed to left shoulder, bilateral wrists, proximal interphalangeal and metacarpophalangeal joints. At age 31 years she was diagnosed with rheumatoid arthritis (RA) by the rheumatology service. At presentation she was on prednisone 5mg daily, methotrexate 17.5mg weekly, status post 2 doses of adalimumab 40mg every 2 weeks. Acetaminophen and diclofenac used as needed. She was previously also on sulfasalazine 1000mg twice daily but was discontinued due to aggressiveness. She was diagnosed with mild intermittent asthma in the past year.

She began to walk at age 4 years. She never attended school, has a vocabulary of 10–12 words, follows basic commands, independently feeds, dresses, and bathes herself. She has a 37 year old brother with mild learning disability; he completed school, works and lives independently. There was no family history of similar congenital defects or autoimmune disorders.

On physical examination, her height was 135 cm and weight was 53 kg. Head was microcephalic with circumference of 51 cm. She was nonverbal and appears to fall under severe-profound intellectual disability. Skin, head and body hair was hypopigmented with a few patches of pigmentation and a large 2×1 cm left neck nevus. Midface is hypoplastic. Eyes were symmetrical with a left limbal dermoid cyst. Mouth was carp like with downturning corners. Residual posterior cleft palate and split uvula were present. Dentition was poor with several teeth broken, missing, or carious. No murmurs were appreciated. On lung exam, bilateral basilar crackles and scattered wheezes were appreciated. There was full range of motion in limbs and normal muscle tone. There was mild tenderness in left shoulder. Surgically corrected left foot noted.

Laboratory results showed positive antinuclear antibody (ANA) at a titer of <1:40, positive rheumatoid factor (RF) at 81.1 IU/mL, negative anti-citrullinated cyclic protein (anti-CCP) at 16AU, elevated erythrocyte sedimentation rate at 42 mm/hr and C-reactive protein at 1.3 mg/dL. Quantitative immunoarray revealed IgA deficiency at 88.5mg/dL, normal IgM and IgG.

Radiographic evaluation revealed osteopenia of left foot, ankle, hands and wrists. Images of knees revealed small left and trace right joint effusion. Transthoracic echocardiogram showed moderate tricuspid valve regurgitation and moderate pulmonary hypertension.

During this admission, high resolution CT chest showed scattered areas of cystic bronchiectasis and bilateral right upper lobe predominant ground glass opacities. She was subsequently treated for bronchiectasis exacerbation with zosyn for a total of 7 days. She improved clinically and was discharged with extensive follow up appointments.

### Cytogenetics

**Figure 1.**
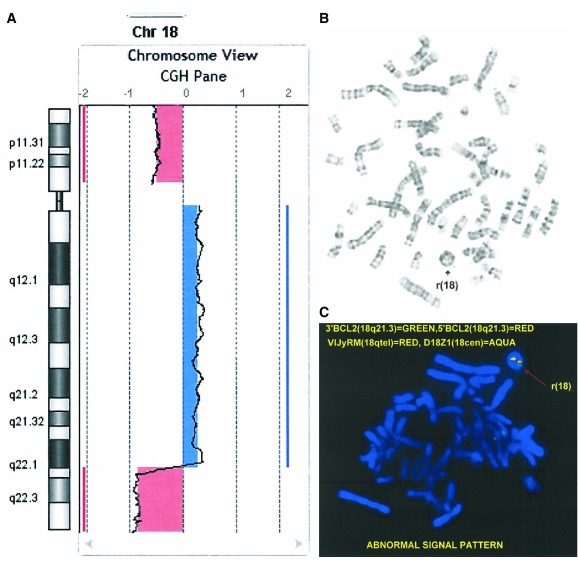
**A**. CGH panel illustrates deletions on distal arms (red) and duplication of proximal long arm (blue). **B**. Karyotype in metaphase showing a ringed chromosome 18. **C**. FISH analysis showing a ringed chromosome 18.

Her primary care physician referred her to the genetics department in our hospital for suspected chromosomal abnormalities. Comparative genome hybridization (aCGH) was done using the custom designed Agilent 44,000 oligonucleotide probes microarray. Probes were placed approximately every 50–100 kb across the entire euchromatic genome with a resolution of 500 kb. The probe density at clinically relevant regions was about 5–10 kb, thus increasing the resolution to 50 kb in targeted regions based on hg19. The aCGH revealed a 14 Mb deletion at 18p11.21-p11.32 (148963-14188180)x1, a 47.8Mb duplication at 18q11.21-q22.1 (18542074-66367715)x3, and a 11.6 Mb deletion at 18q22.1-q23 (66377285-78010032)x1 ( Figure 1a). Standard chromosome analysis which involved growing of lymphocytes obtained from peripheral blood in RPMI culture media supplemented with L-glutanin and Phytohemagglutanin (PHA) and antibiiotics for 72 hours, followed by mitotic arrest by adding colchicine and hypotonic treatment and fixation of cells in 3:1 methanol:acetic acid. Chromosome slides were prepared 24 hours later, and then GTG banded using Trypsin and Giemsa. Chromosome analysis was performed using the Ikaros Software (Metasystem, Germany). Of the 20 cells analyzed, 14 cells showed a ring chromosome 18 which was determined to be of chromosome 18 origin, and 4 cells showed a normal female chromosome complement. The dicentric ring was seen as a single non-clonal anomaly that was not confirmed by FISH analysis and hence omitted in the discussion of this manuscript. Fluorescence *in situ* hybridization Analysis (FISH) was performed to confirm the chromosome analysis findings. Briefly, slides aged for 24 hours were pretreated in 2XSSC. ThermoBrite (Vysis Inc., IL, USA) was utilized for the denaturation (73 C for 6 minutes) of the BCL2 and D18Z1 (centromere) probes followed by co-hybridization of probes to chromosomal DNA on the slides. Slides were then subjected to post-hybridization washes. Image analysis was performed using the Metasystem Ikaros Software (Metasystem, Germany). FISH analysis confirmed that 75% of the cells were ring 18 in origin with the presence of 2 BCL2 signals and one centromere 18 signal ( Figure 1c). The 18 centromere signal in the picture is obscured due to its close proximity with the breakpoints close to the BCL2 DNA probe.

## Discussion

The described chromosomal abnormalities are most likely *de novo*, since maternal analysis was normal and her father was normal in appearance and health. Furthermore, ring chromosomes usually arise *de novo*, with only 1% inherited ^10^. Of the inherited cases, 90% are maternal since the presence of a ring blocks spermatogenesis and induces infertility in males ^12, 13^.

Consistent with previously reported cases of r(18) and RA (see Table 1), our patient had many of the characteristics associated with both 18p- and distal 18q- syndrome. The features shared between the two conditions include intellectual disability, short stature, microcephaly, IgA deficiency, autoimmune disorders (RA, vitiligo, hypothyroidism), and likely conductive hearing loss. Features specific to 18p- include excessive dental caries, while those specific to distal 18q- include cleft palate, carp shaped mouth, and clubfoot. Duplication did not appear to cause any distinguishing Edwards syndrome manifestations such as clenched fist, rocker bottom feet, severe organ involvement, and failure to thrive.

**Table 1.** Clinical Comparison of Proband with some Previously Reported 18p- and 18p+ cases with Rheumatoid Arthritis (RA).

Authors	Syndrome	Sex	Age of onset	Joint involvement	Extra-articular manifestations	Lab studies	Other immunological and endocrine problems	Major abnormalities	Developmental problems
Finley *et al.* ^7^ (1972)	18p-	F	9 months	Pain/swelling in wrists, R knee, R ankle; contractures in elbows, knees, hips	Fever, rash, hepatospleno-megaly	+ANA	-	Short stature, hypertelorism, epicanthal folds, flat nasal ridge, short fifth finger, elevated triradius	Mild MR, delayed speech
Petty *et al.* ^8^ (1987)	r(18)	M	11 years	Pain/swelling in knees, ankles; contractures and effusions in knees X-ray: wnl	-	- ANA + RF	IgA deficiency	Tetralogy of Fallot, microcephaly, short stature, midface dysplasia, nystagmus, malabsorption	Severe MR
	Interstitial 18q- **	F	5.5 years	Pain/swelling in knees, R wrist, ankles; effusions in knees X-ray: wnl	Erythematous macules, urticaria	- ANA - RF	Reduced IgG and IgM	Short stature, hypertelorism, flat nasal bridge, broad palate, bifid uvula, external auditory canal atresia, large mouth, unilateral simian crease, short/broad hands	Moderate MR
Hansen *et al.* (1994) ^9^	Distal 18q-	M	4 years	Pain/swelling in knees, L ankle; effusions in knees; contracture L knee; XR: TMJ arthritic changes, L knee >subchondral erosions, L patella enlargement	Uveitis	+ANA - RF	Elevated IgG and IgM	Hypospadias, umbilical hernia, cleft left palate, hypertelorism, atretic ear canals, severe hearing loss, hypotonia, joint hypermobility, syndactylia of second and third toes	Normal IQ range with learning difficulty, delayed speech, attention, visual- spatial orienting, fine and gross motor skill difficulties
Czakó *et al.* (2002) ^10^	Translocation:18p-, 20p trisomy	F	6 years	Large joints, small finger joints	-	+ESR +CRP + Waaler Rose test	Milk protein intolerance	Oval flat face, upslanting palpebral fissures, periorbital fullness, hypoplastic midface, flat nose, down turned corners of mouth, muscular hypotonia	Severe psychomotor retardation
Rosen *et al.* (2004) ^6^	Distal 18q-	F	8 years	Pain/swelling, effusions in knees, R SCJ X-ray: knee effusions MRI: R SCJ synovitis	-	+ ANA - RF DRB*11 allele	Elevated IgG	ASD, short stature, left aural atresia, right external auditory canal atresia, prominent nasal pyramids, hypoplastic alae nasi, broad mouth, thin upper lip, short philtrum, joint hypermobility	Normal IQ range with learning difficulty
Recacalti *et al.* ^5^ (2010)	18p-	F	<5 years?	?	?	?	-	Seizures, microcephaly, midline anomaly (ectopic neurohypophysis) growth retardation, blue sclera, sparse hair, upslanting palpebral fissures, epicanthal folds, high arched palate, low set, ears, micrognathia, retrognathia, excessive caries, cupid bow lips, long philtrum, short neck, short and broad hallux, sacral dimple, hypotonia	MR, speech delay, psychomotor retardation
Our case	r(18)	F	19 years	Pain/swelling in L shoulder, knees, wrists, hands X-ray: knee effusions; osteopenia in L foot, ankle, hands, wrists	ILD?	+ ANA + RF - CCP +ESR +CRP	IgA deficiency, hypothyroidism, vitiligo	Short stature, microcephaly, cleft palate, bifid uvula, midface hypoplasia, carp- like mouth, excessive dental caries, hearing loss, club foot	Severe MR

+ = present, - = absent, ? = not reported; F = female, M = male, R = right, L = left; ANA = antinuclear antibodies, RF = rheumatoid factor, CCP = cyclic citrullinate peptide; ASD = atrial septal defect, IQ = intelligence quotient, MR = mental retardation ** Possible drug related etiology, occurred after trimethoprim sulfamethoxazole use with intermittent reoccurrences

The unique feature of our patient was her late onset of RA compared to the early JRA previously reported ^5– 9, 10^. Our patient was RF positive, anti-CCP negative and met 9/10 of the 2010 American College of Rhematology (ACR) Clinical Classification Criteria for RA ^7^. To the best of our knowledge there have been seven reported, and six published, cases of RA associated with chromosome 18 abnormalities ( Table 1). Daentl first mentioned an unpublished case of JRA in a patient with 18p- and IgA deficiency ^8^. In the first published case, Finley *et al.* reported a 9-month-old female with 18p-, swelling and contractures in many joints, fever, rash, and hepatosplenomegaly, consistent with JRA ^7^. JRA has subsequently been associated with cases of 18q- as well ^6, 8, 11^.

The exact genes involved with RA and autoimmune disease development in chromosome 18 abnormalities are still not well defined. There is a suggested link between PTPN2 (protein tyrosine phosphatase non-receptor type 2), located at 18p11.2-11.3, and RA and T1DM ^14, 15^. Genome wide studies have shown evidence for the association of the PTPN2 locus with RA susceptibility in both Japanese and European populations ^14, 16^. A case similar to ours was reported by Jain *et al.* with *de novo* r(18), del(18q23-18qter) and del(18p11.3-18pter) associated with hyperthyroidism, T1DM, vitiligo, and IgA deficiency, but not RA ^4^. Their case had a more distal deletion that likely spared PTPN2. Another autoimmune critical region was proposed on 18p, with molecular breakpoints at 12,316,423-1,231,7830; interestingly, PTPN2 is not in this region ^15^. The genetic basis of autoimmune disease is not as well established in 18q- compared to 18p-. On the long arm, Merriman *et al.* proposed a locus at 18q12-21 that influences development of autoimmune diseases ^17^. Another gene of interest is NFATc1 (nuclear factor of activated T cells) at 18q23, implicated in maintaining the programmed death receptor (PD-1) and ligand (PD-L) pathway that is essential for regulatory T cells to terminate immune responses and protect against autoimmunity ^18, 19^. It is difficult to establish a definitive genotype-phenotype association, but it appears plausible that this proposed autoimmune critical region and PTPN2 on the short arm, as well as NFATc1 on the long arm may play a part in the autoimmune diseases seen in our patient.

Overall, adult RA has a poorer outcome compared to JRA ^20^. Mortality rates in RA patients are increased due to medication related infections, gastrointestinal bleeding as well as extra-articular pulmonary, renal disease, and cardiovascular manifestations ^20^. Our patient was also found to have ILD, bronchiectasis, and pulmonary hypertension. To the best of our knowledge, there is no known connection between interstitial lung pathologies and chromosome 18 abnormalities. However, ILD and bronchiectasis are known extra-articular manifestations of RA. In a population study, the lifetime risk of developing ILD was 7.7% for RA patients and 0.9% for non-RA subjects ^21^. The classic presentation is a reticular, reticulonodular or honeycomb pattern in the lung bases.

The patient was also on several medications known to cause drug-induced interstitial lung disease (DI-ILD) including: methotrexate, adalimumab, sulfasalazine, and diclofenac ^22^. Our report describes the first case of late onset RA associated with mosaic 18p-, 18q- and dicentric r(18). The complex rearrangements were detected by aCGH, karyotype and FISH. Her syndrome has features of both 18q- and 18p-, including multiple autoimmune disorders that support the idea of genetic loci on chromosome 18 playing a role in disease expression. Additionally, the finding of ILD - whether caused by RA, drug exposure, or an unexplored linkage - is an important condition to be aware of in patients with chromosome 18 abnormalities and autoimmune diseases.

## Consent

Written and informed consent was obtained from the mother, who was the designated health care proxy prior to publishing this case report, for publication of any potentially identifiable clinical data that may be associated.
