# Supplementary material for: Rheumatoid arthritis in an adult patient with mosaic distal 18q-, 18p- and ring chromosome 18
Source: F1000Res. 2018 Feb 28;6:1940. Originally published 2017 Nov 2. [Version 2] doi: 10.12688/f1000research.11539.2 (PMC5854985; doi:10.12688/f1000research.11539.2)
Supplement: Peer review history

# Peer review: Referee response for version 2

**DOI:** 10.5256/f1000research.15249.r31262

**Published:** 2018-03-14

**Authors:** Jannine D. Cody

**Affiliations:** 1 Department of Pediatrics, University of Texas Health Science Center at San Antonio, San Antonio, TX, USA

The additional information has made this acceptable for publication

I have read this submission. I believe that I have an appropriate level of expertise to confirm that it is of an acceptable scientific standard.

# Peer review: Referee response for version 2

**DOI:** 10.5256/f1000research.15249.r31261

**Published:** 2018-03-06

**Authors:** György Kosztolányi

**Affiliations:** 1 Department of Medical Genetics, Clinical Center, University of Pécs, Pécs, Hungary

I have no additional remark.

I have read this submission. I believe that I have an appropriate level of expertise to confirm that it is of an acceptable scientific standard.

# Peer review: Referee response for version 1

**DOI:** 10.5256/f1000research.12465.r27903

**Published:** 2017-11-27

**Authors:** Jannine D. Cody

**Affiliations:** 1 Department of Pediatrics, University of Texas Health Science Center at San Antonio, San Antonio, TX, USA

This manuscript describes a single 32 year old woman with Ring 18 and rheumatoid arthritis. Overall the manuscript is well written, however there are several items that need further work before publicaiton.

1. Most of the literature on Ring 18 has been omitted. The paper would benefit from a better review of Ring 18 as opposed to mostly 18p- and 18q-. Has the lung disease eve been reported?
2. The methods and data that determined the actual chromosome content are missing. The percent that each cell type is present in the blood as well as the FISH studies definitively demonstrating each should be included. From the text it is not clear if the “duplication of the long arm” is present as a ring or an isochromosome. The aCGH showing net copy number suggests that not all of the long arm is duplicated since a larger proportion of cells have an 18q terminal deletion than have an 18p terminal deletion. What percent of the cells actually have a ring chromosome?
3. The interpretation of the FISH data needs to be described more fully. Why are there two BCL2 probes? I don’t see a D18Z1 signal?
4. The table needs to be made more clear in its title that it only includes RA cases and not all Ring 18 or 18q-, 18p- cases (at least I think that is the intent).
5. In the first paragraph of the discussion, the last sentence needs a reference.

I have read this submission. I believe that I have an appropriate level of expertise to confirm that it is of an acceptable scientific standard, however I have significant reservations, as outlined above.

# Peer review: Referee response for version 1

**DOI:** 10.5256/f1000research.12465.r27904

**Published:** 2017-11-23

**Authors:** György Kosztolányi

**Affiliations:** 1 Department of Medical Genetics, Clinical Center, University of Pécs, Pécs, Hungary

This is a well documented case report which may contribute to our knowledge on the clinical consequences of chromosome 18 abnormalities. I suggest to accept the MS for publication on condition that the authors consider a major and some minor remarks for modification.

**Major remark**

 The routine cytogenetics description is too short. Nothing is written about the percentages of the "mosaic cell lines” (as the authors write), although one of the unique characteristics of ring chromosomes is their dynamic nature. As a result of mitotic difficulties, a ring chromosome is subject of additional cytogenetic mutations, resulting in continuous generation of secondary aneuploidy cells. Accordingly, this dynamic mutations series may manifest themselves as "mosaic cell lines”, however, the survival of such cells as cell line, as well as the explanation of their presence being "cell lines” is questionable. I would recommend to refer to this wildely accepted explanation for the presence of differentially shaped chromosomes in patients with ring chromosome, at least as an alternativ possibility. (e.g. Kosztolányi G: Does "ring syndrome" exist? Hum.Genet. 1987; 75:174-179)  

**Minor remark**

 Some peculiarity of the case should be highlighted with more emphasis. E.g., Detecting a chromosome abnormality in a patient at the age of 32 is rare – it should be highlighted in the paper. Also the extreme severity of the somatic and mental underdevelopment should be pointed on abstract.

I have read this submission. I believe that I have an appropriate level of expertise to confirm that it is of an acceptable scientific standard, however I have significant reservations, as outlined above.
